# Dental Pathology

**Published:** 1873-12

**Authors:** W. L. Younger


### ARTICLE II.

*Dental Pathology.*—BY W. L. YOUNGER, M. D.

Read before the California State Dental Association.

The diseases of the mouth and dental organs are now being so well defined, and becoming tractable to treatment, and the necessity so apparent for preserving these organs of our blood, so important to health, beauty and comfort, that the dentist who does not put forth every effort that skill and ingenuity can devise to render extraction unnecessary, is unworthy of his profession, and should be ostracised from our respect and society. I now beg leave to present a few cases, hoping that a consideration of them may be of service.

A gentleman came to me to have his teeth extracted. He had been recommended to this by his physician, and some of the best dentists, both here and in the Eastern States; yet his teeth were perfectly sound. Two of them only had been decayed and these were well filled. There was not a single devitalized tooth in his head. But his teeth were loose and painful; the gums had receded fully one-half the length of the roots, and were swollen and spongy; pressure on them caused the exudation of yellowish pus from their margins, and there was intolerable *fœter* of the breath. The gentleman noticed the commencement of the disease fifteen years before; but, on application to the best authorities, he was told nothing could be done for them but extraction. He preferred to keep them as long as possible, and when he could bear them no longer he came to me. I passed some delicate hoe-shaped instruments up along the roots, as far as gentle pressure would carry them, and then holding the teeth with the unoccupied hand, to keep them from being pulled out, by gentle traction I disengaged black tartar, that had caused all the mischief, and which clung with considerable tenacity to the roots. I then scraped the carious edge of the alveolar process, pointed it with iodine, as also the gums, and prescribed an astringent wash. In one week he returned. Upon examination, I found, first, that the gums had resumed nearly a normal appearance; second, that the fearful *fœter* of the breath had almost disappeared; third, that there was but slight discharge of pus from a few of the teeth only; and, fourth, they were firmer in their sockets and less sensitive to pressure. I passed the hoes along the roots again, to assure myself that there was no more tartar remaining on them, and continued the treatment with iodine and the astringent wash. I further ordered him to press the gums with his fingers, commencing at a point over the roots as high as the attachment of the buccal muscles would let him, and pressing toward the crown of the tooth, but not over the margins. By this means, when I last saw him, he had reduced the exposed part of the root about two lines: he was discharged in six weeks, cured.

There was one case that puzzled me very much. The upper front teeth including the canines and first biscuspids, were very sensitive to pressure, yet there was no tartar on the roots, the gums had not receded, and, with the exception of a slightly deeper hue, were normal in appearance; neither was there any caries of the teeth; yet they were not as firm as they should have been, but their lack of firmness was not by any means remarkable. Counter-irritants and bleeding of the gums failed to give him any permanent relief, and it was not until a very serious nasal catarrh, with which the gentleman had been affected, had been cured, that the abnormal sensitiveness of the teeth disappeared, and they became again useful and comfortable organs. In this case the disease was not in the mouth at all, it was merely the extension of the irritation, caused by the catarrh, from the floor of the nose to the dome of the sockets, and serves to show that when the dentist can not find the disease in the mouth, or dental organs proper, he must look beyond, to the contiguous parts. And this proves, that for a dentist to be thoroughly qualified to practice his profession, he should have a much more thorough medical training than is usually given him, or he thinks necessary. But I will pass on to other cases.

A Mr D——, 22 years of age, from Acapulco, presented himself to me last November. The appearance of his face showed at once the ravages of a dental fistula. The history of his case was this: Six months previously he was attacked with a severe throbbing pain, and great swelling in the region of the angle of the left inferior maxilla. A quantity of pus gathered, which at last found exit, externally by an opening in the cheek, a half inch over the notch of the facial artery. Being of a scorbutic diathesis, his physician treated him for scrofula, and it was not until the end of of the six months, when the discharge continuing obstinate, and the patient getting no better, that he commenced to suspect a slightly decayed second molar as the cause of the mischief, and ordered him to San Francisco to consult a dentist. Upon an examination, I found the cause of the trouble to be nothing but a retained wisdom tooth, one of the posterious knobs of which had only pierced the gum. The anterior knobs had evidently, in the process of growth upward, caught in the neck of the second molar, while the roots were held in the angle of the maxilla. The face showed a great depression in the region of the fistulous opening and a prominent tumefaction immediately over it. I extracted the tooth, and in doing so brought out a great deal of very fœtid pus, full of broken-down particles of necrosed bone. On probing the wound made by the extraction, I was astonished at the great destruction that had occured in the bony formation of the jaw. I do not think there was over half an inch in depth of healthy bone left in the angle. After scraping off the exposed surface of bone in the wound, and detaching little pieces that were sticking to the fleshy walls, and rinsing the part well with water, I bathed the cavity with carbolic acid and iodine, and introduced a pleget of charpie, saturated with these agents, to the bottom of the excavation, to prevent the closing of the wound and force the granulations to rise from the bottom. The discharge through the face ceased almost synchronously with the extraction of the tooth, and the part quickly healed up. I then advised rubbing and working the tissues of the depressed portion of the face, so as to thicken the integuments. And, in the course of a few weeks, I had the satisfaction of seeing the depressed portions fill out nearly to the condition of the opposite side of the face, the tumefactions subside, and the wound fill up with healthy granulations to within a quarter of an inch of the surface, when his stay here being no longer necessary, he departed for his home in Mexico.

Another case is that of a lady, where, in consequence of the necrosis of a superior lateral, a destruction of bone had occurred, extending from the external surface of the superior maxilla to its palatial face, involving the antrum highmorianum. Bad as the tooth was, it yet acted as a tent-pole to the gum ; and its extraction would have produced a fearful deformity, that might defy the skill of a plate-worker to remedy. I set immediately to work to cure the root, and then, making a free crucial incision over the apex of the tooth, scraped off all extraneous and diseased matter. I then injected as in the previous case, carbolic acid and iodine, and occasionally a strong solution of chlor. of zinc, and kept open the wound by a pleget of charpie. Finding a month ago that there was no pus discharged, and the appearance of the mouth was that of health, I caused the injection to be simply of water, three times a day, a little salt being occasionally added to the liquid. I believe that in the course of time all the destroyed space will be restored by healthy tissue.

In the case of a Mrs. B., I cured an obstinate root of ulceration that I had treated with failure by the usual methods, by applying through the canal of the root, sulphuric acid, by means of a little cotton on a fine gold wire, and after the acid had its effect, neutralizing it with liq. ammonia, applied likewise with cotton. Two applications had the desired effect, and now, at the end of eight months, I find the tooth, to all appearances, in as good condition as if it had never been guilty of an ulceration.

Three months ago a Mr. P. came to me with a highly sensitive first left superior molar. The attrition consequent on mastication had worn off the enamel over several points in the grinding surface, the internal or lingual root was exposed over one half of its length, by the recession of the gum in consequence of a disease of the alveolus, occasioned by the presence of tartar, and all these parts, that were denuded of enamel and of gum, were exquisitely sensitive, not only to cold and heat, but to contact with food and the air of respiration. The tooth had been suffering from this exalted sensibility for some time, and had become at last so painful as to be unendurable. The gentleman had already lost the central and the corresponding molar of the opposite side, from this same cause—the different dentists to whom he had applied knowing of no better cure than extraction. I applied the granulated chloride of zinc to the sensitive parts, allowing it to deliquese with the moisture of the breath, in order to impress the parts with its full strength; but, notwithstanding the repeated applications of this salt, the tooth continued as sensitive as ever. I tried counter irritants, imagining the unusual sensibility due to internal irritation, but without the desired effect. At last, I drilled a cavity in the tooth, and put the arsenious application in common use. This was effectual. I then drilled further, devitalized the pulp, extracted it, and filled the tooth, which has been comfortable ever since; the only trouble now remaining being the diseased alveolus, mentioned before, and which is now about cured.

I hope at the next meeting to be able to report the successful termination of a case, that I have already commenced treating, the particular interest of which is due to the vastness of the lesions, it involving nearly the whole of the left superior maxillary and contiguous parts.

A freak of dentition has just come to my notice, showing the complete independence of the permanent from the deciduous teeth. A child that had never had the four superior temporary incisors, at the eighth year commenced cutting the corresponding permanent ones, and has them now in the proper place, well formed and regular.

